# The synergistic effect of diabetes mellitus and low high-density lipoprotein cholesterol on the prediction of pneumonia in peritoneal dialysis patients: a multicenter retrospective study

**DOI:** 10.3389/fnut.2025.1739917

**Published:** 2026-01-13

**Authors:** Sijia Shang, Xing Zhang, Xiaojiang Zhan, Na Tian, Yueqiang Wen, Xiaoyang Wang, Xiaoran Feng, Qian Zhou, Xianfeng Wu, Qingdong Xu, Guojun Shi, Yijia Zheng, Ning Su

**Affiliations:** 1 Department of Hematology, The Sixth Affiliated Hospital, Sun Yat-sen University, Guangzhou, China; 2 Biomedical lnnovation Center, The Sixth Affiliated Hospital, SunYat-sen University, Guangzhou, China; 3 Department of Nephrology, The First Affliated Hospital, Nanchang University, Nanchang, China; 4 Department of Nephrology, General Hospital of Ningxia Medical University, Yinchuan, China; 5 Department of Nephrology, The Second Affliated Hospital, Guangzhou Medical University, Guangzhou, China; 6 Department of Nephrology, The First Affiliated Hospital of Zhengzhou University, Zhengzhou, China; 7 Department of Nephrology, Jiujiang No. 1 People’s Hospital, Jiujiang, China; 8 Department of Medical Statistics, Clinical Trials Unit, The First Affiliated Hospital, Sun Yat-sen University, Guangzhou, China; 9 Department of Nephrology, Shanghai Jiao Tong University Affiliated Sixth People's Hospital, Shanghai, China; 10 Department of Nephrology, Jiangmen Central Hospital, Jiangmen, China; 11 Department of Endocrinology & Metabolism, Medical Center for Comprehensive Weight Control, The Third Affiliated Hospital of Sun Yat-sen University, Guangzhou, Guangdong, China; 12 Guangdong Provincial Key Laboratory of Diabetology & Guangzhou Municipal Key Laboratory of Mechanistic and Translational Obesity Research, The Third Affiliated Hospital of Sun Yat-sen University, Guangzhou, Guangdong, China

**Keywords:** diabetes mellitus, HDL-C, infection, peritoneal dialysis, pneumonia

## Abstract

**Background:**

Patients undergoing peritoneal dialysis (PD) often exhibit abnormal glucose and lipid metabolism. Pneumonia is an important infectious complication in PD patients. Diabetes mellitus (DM) and low levels of high-density lipoprotein cholesterol (HDL-C) are risk factors for pneumonia. This study aimed to investigate the relationship between the co-existence of DM and low HDL-C and the occurrence of pneumonia in PD patients.

**Methods:**

This multicenter retrospective study was conducted across five PD centers from January 1, 2008, to December 31, 2020. The primary outcome was new-onset pneumonia. Patients were divided into four groups based on the presence of DM and low HDL-C. Cox regression analysis was performed to evaluate the association between the co-existence of DM and low HDL-C and the development of pneumonia.

**Results:**

A total of 1,293 PD patients were included in this study. During a median follow-up period of 53 months, pneumonia occurred in 137 patients (10.6%). Multivariable Cox regression analysis demonstrated that patients with coexisting DM and low HDL-C had a significantly increased risk of pneumonia compared to those without either condition (HR = 2.252, 95% CI, 1.232–4.115). Consistent results were observed in subgroup analyses stratified by age, sex, and smoking history.

**Conclusion:**

The concurrent assessment of DM and low HDL-C may help identify high-risk individuals for pneumonia and guide the management strategies for PD patients.

## Introduction

The prevalence of chronic kidney disease (CKD) is progressively increasing, driven by global population aging and lifestyle changes (1). Peritoneal dialysis (PD) is a prevalent renal replacement therapy, accounting for 11% of all dialysis (2). PD patients demonstrate elevated infection susceptibility, with infections constituting the second most frequent etiology of hospitalization and mortality. Pulmonary infections account for approximately 25% of all infection-related deaths in PD patients. Studies have shown that the mortality rate following pulmonary infections is 14–16 times higher in dialysis patients compared to the general population (3, 4).

The prevalence of diabetes mellitus (DM) has increased steadily, with diabetic nephropathy (DN) emerging as a primary cause of end-stage renal disease (ESRD) in numerous countries. The association of DM with an elevated risk of developing infectious diseases is well-documented. Studies have shown that diabetic patients have a 1.75 times higher risk of developing pneumonia compared to non-diabetic individuals (5). In a separate study, a similar conclusion was corroborated, revealing that patients undergoing continuous ambulatory peritoneal dialysis (CAPD) combined with DN had a significantly higher risk of pneumonia compared to those without DN (6).

Dyslipidemia is common in patients with PD, typically presenting with elevated triglyceride levels and reduced high-density lipoprotein cholesterol (HDL-C) (7). This specific lipid profile—high triglycerides and low HDL-C—affects approximately 25–50% of PD patients (8). Extensive research has established the link between low HDL-C and atherosclerosis. Furthermore, growing evidence suggests that the magnitude of lipid level alterations associates with infection severity (9, 10). For every 10 mg/dL rise in baseline HDL-C, the risk of hospitalization for pneumonia decreased by 10% (11). Despite the high prevalence of glucose and lipid metabolism disorders in PD patients, research has not evaluated how combined glucose-lipid metabolic disorders affect pneumonia risk. Determining whether DM coexisting with low HDL-C increases pneumonia susceptibility would enhance infection risk stratification in PD patients. The purpose of this study was to explore the relationship between DM coexisting with low HDL-C and new-onset pneumonia in PD patients.

## Methods

### Study design and participants

This was a multicenter retrospective study conducted in five PD centers from January 1, 2008 to December 31, 2020. Exclusion criteria comprised age <18 years, follow-up duration < 3 months, or unavailable HDL-C measurements. This investigation adhered to the ethical principles of the Declaration of Helsinki and received formal approval from the Institutional Review Board at Sun Yat-sen University’s Sixth Affiliated Hospital (No. 2021SLYEC-177). Written informed consent was obtained from all enrolled patients during hospital admission.

### Data collection

All patients underwent CAPD treatment. Demographic and laboratory data were systematically extracted from the institution’s electronic medical records system. All study data were collected within three months of PD treatment. Demographic parameters comprised age, sex, body mass index (BMI), smoking/alcohol use history, and pre-existing comorbidities [cardiovascular disease (CVD), stroke, DM, hypertension]. Laboratory parameters encompassed complete blood count indices (leukocytes, hemoglobin), metabolic markers (albumin, fasting glucose, creatinine, uric acid, urea nitrogen), lipid profile components (total cholesterol, triglycerides, HDL-C, LDL-C), and electrolyte levels (calcium, phosphorus, potassium). The total cholesterol, triglyceride, HDL-C, and LDL-C levels of all patients were obtained following a 12-h strict fasting period. BMI was computed using the standard formula: weight (kg) divided by height squared (m^2^). DM was defined as (1) HbA1c ≥ 6.5%, (2) fasting plasma glucose ≥ 7.0 mmol/L, (3) 2-h plasma glucose ≥ 11.1 mmol/L during an OGTT, (4) in a patient with classic symptoms of hyperglycemia or hyperglycemic crisis, a random plasma glucose 11.1 mmol/L. Diagnostic criteria 1–3 require confirmation through repeat testing in the absence of unequivocal hyperglycemia (12). The DM status in this study was based on patients’ pre-existing DM diagnoses documented in their medical records. The disease type of all DM patients in this study was type 2 DM. Hypertension was recorded if the patient was taking antihypertensive medication or had two blood pressure measurements ≥140/90 mmHg (13). The range of activity of HDL-C in PD patients is still not entirely comprehended. Utilizing the receiver operating characteristic (ROC) curve, patients were categorized into two groups: low HDL-C levels and high HDL-C levels. Smoking history was defined as at least one cigarette per day, and drinking history was defined as > 20 g of ethanol per day. Each PD center has two professionally trained graduate students for data collection and collation.

### Outcomes and follow-up

The primary outcome of this study was new-onset pneumonia. The diagnosis of pneumonia is performed by a professional respiratory physician based on the patient’s symptoms, such as cough, dyspnea, and fever, the results of laboratory tests, and the presence of new pulmonary infiltrates on the chest X-ray or CT (14). The pneumonia diagnosed in this study is primarily community-acquired pneumonia. Other participants were censored when they died, transferred to hemodialysis, transferred to another centers, transferred to kidney transplant, or censoring on March 1, 2021.

### Statistical analysis

All continuous variables were tested for normality. All continuous variables were non-normally distributed, represented by median and IQR (P25- P75). The classification variable is expressed in number (percentages). HDL-C was brought into ROC curve analysis and the cut-off value was determined according to the Youden’s J index. All patients were divided into four groups: Group 0 (patients without DM and low HDL-C), Group 1 (patients with only DM), Group 2 (patients with only low HDL-C), Group 3 (patients with the coexistence of DM and low HDL-C). The differences of continuous variables between groups were tested by one-way ANOVA or Kruskal-Wallis test. Comparison of categorical variables by Chi-square test. Univariate Cox regression analysis was used to explore the risk factors of new-onset pneumonia in PD patients. The Kaplan–Meier curve was used to generate the cumulative event curves among the four groups during follow-up. Log-rank test was applied to compare survival between groups. A stratified Cox proportional hazards model according to the center was used to estimate HR with 95% CI. Model 1: unadjusted; Model 2, Model 1 plus age, sex, BMI, smoking history, pre-existing stroke, pre-existing CVD, hypertension; Model 3, Model 2 plus hemoglobin, serum albumin, fasting blood glucose (FBG), triglyceride, LDL-C, creatinine, uric acid, serum potassium, serum calcium. This study conducted a subgroup analysis by sex, age and smoking history, and interaction tests were conducted to examine the coexistence of diabetes and low HDL-C and the interaction between various causes and pneumonia. All statistical analyses were performed with SPSS (version 25) and GraphPad (version 8.0.2). *p* values were bilateral, and *p* < 0.05 was considered to be statistically significant.

## Results

### Baseline characteristics of participants

A total of 1,417 patients were collected in four PD centers. 124 patients were excluded for the following reasons: age < 18 years (*n* = 11), maintenance dialysis time less than 3 months (*n* = 70), lack of HDL-C data (*n* = 43). Finally, 1,293 PD patients were included in the statistical analysis.

**Figure 1:**
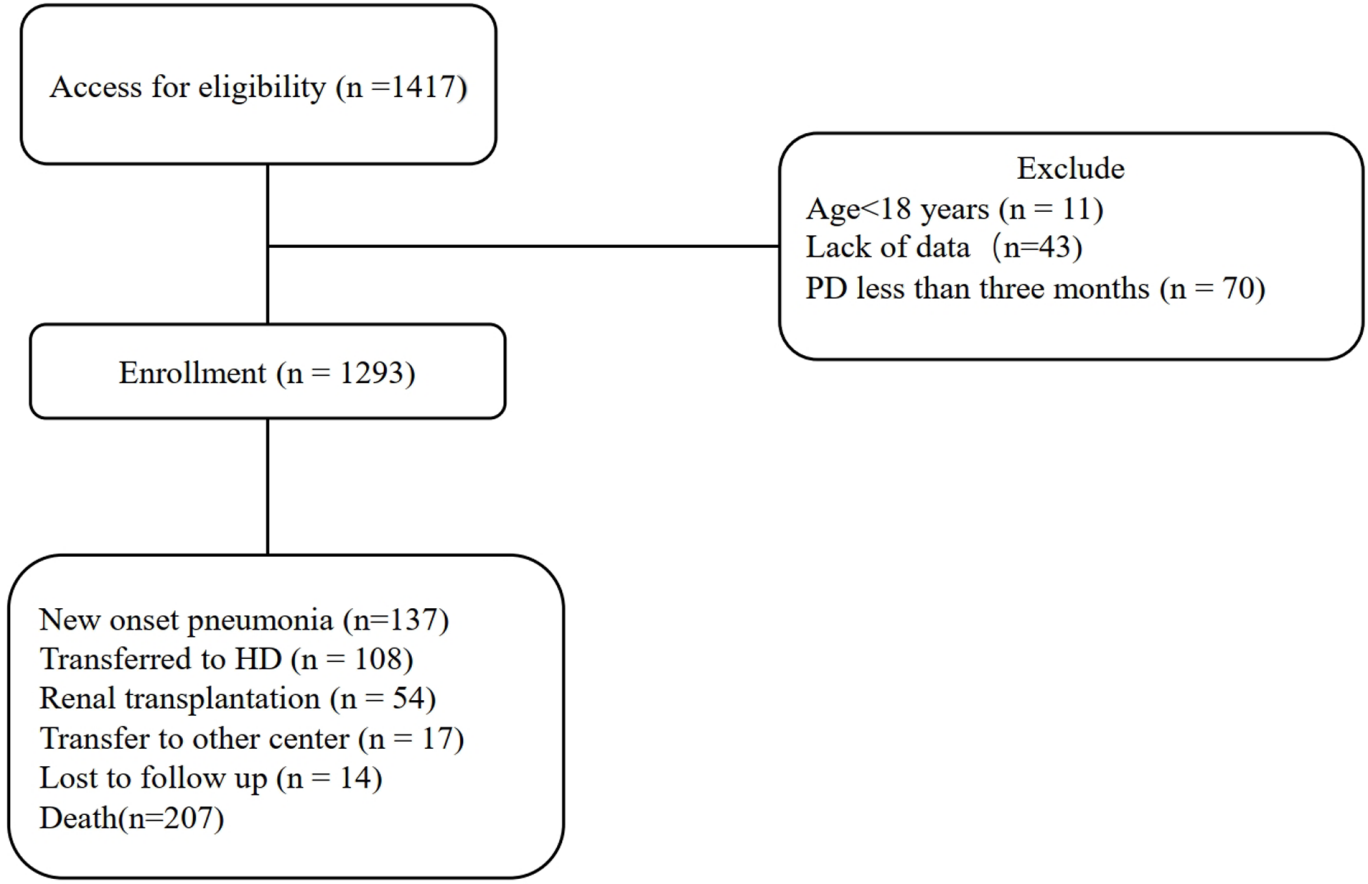
The flow chart shows the exclusion and selection of patients. PD, peritoneal dialysis; HD, hemodialysis.

**Figure 2:**
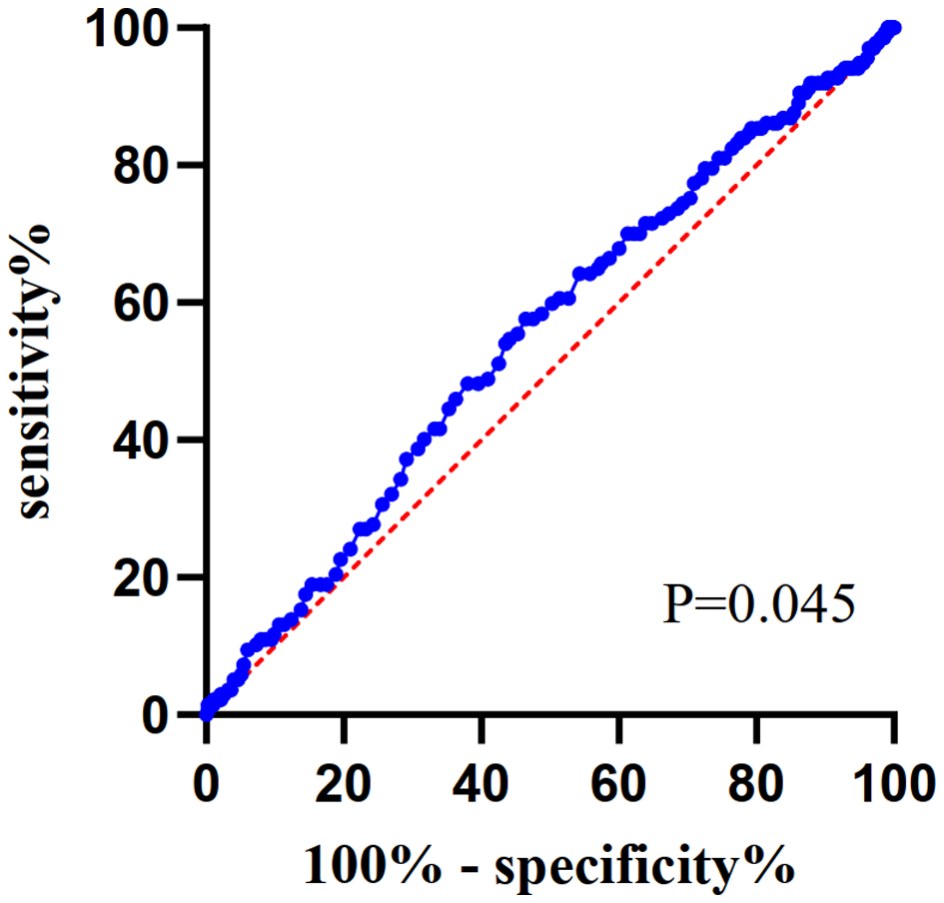
Receiver operating characteristic (ROC) curve of HDL-C.

During the median follow-up period of 53 months, a total of 137 (10.6%) patients developed pneumonia, 207 (16%) patients died, 108 (8.4%) patients transferred to hemodialysis, 54 (4.2%) patients transferred to kidney transplantation and 17 (1.3%) patients transferred to other centers (Figure 1). ROC curve analysis identified an HDL-C cut-off value of 1.075 mmol/L (Figure 2). This study identified that 13.3% of patients had coexisting DM and low HDL-C, 11% had DM without low HDL-C, and 34.4% of patients had low HDL-C without DM. The incidence of new-onset pneumonia in Groups 0, 1, 2 and 3 were 6.5%, 16.2%, 8.8% and 23.3% (Table 1).

**Table 1:** Demographic and baseline clinical data.

Variables	Total (*n* = 1,293)	Group 0 (*n* = 535)	Group 1 (*n* = 142)	Group 2 (*n* = 444)	Group 3 (*n* = 172)	*p*-value
Demographics						
Age (years)	55 (43–65)	51 (39–63)	61 (52–68)	53 (41–65)	61.5 (53–69.75)	<0.001
Male [*n* (%)]	741 (57.3%)	252 (47.1%)	87 (61.3%)	290 (65.3%)	111 (64.9%)	<0.001
BMI (kg/m^2^)	22.48 (20.42–24.46)	21.46 (19.65–23.24)	23.83 (21.65–25.23)	22.61 (20.72–24.84)	24.02 (21.78–26.92)	<0.001
Smoking history [*n* (%)]	101 (7.8%)	23 (4.3%)	16 (11.3%)	41 (9.2%)	21 (12.2%)	0.001
Drinking history [*n* (%)]	36 (2.8%)	7 (1.3%)	5 (3.5%)	18 (4.1%)	6 (3.5%)	0.056
Comorbid						
Pre-existing stroke [*n* (%)]	56 (4.3%)	12 (2.2%)	12 (8.5%)	14 (3.2%)	18 (10.5%)	<0.001
Pre-existing CVD [*n* (%)]	293 (22.7%)	107 (20%)	50 (35.2%)	69 (15.5%)	67 (39%)	<0.001
Pre-existing hypertension [*n* (%)]	751 (58.1%)	267 (49.9%)	117 (82.4%)	222 (50%)	145 (84.3%)	<0.001
Laboratory variables						
Leukocyte (10^9^ /L)	6.40 (5.34–7.72)	6.31 (5.09–7.17)	6.53 (5.56–8.39)	6.40 (5.42–7.64)	7.22 (6.03–9.27)	<0.001
Hemoglobin (g/L)	103 (88–118)	106 (91–121)	102 (88–118)	99.5 (85–116)	103.5 (90–115)	0.001
Serum albumin (g/L)	34.00 (30.60–37.15)	34.30 (31.10–37.90)	31.50 (27.48–34.23)	34.65 (31.9–38)	32.05 (28.39–35.48)	0.323
Fasting blood glucose (mmol/L)	4.96 (4.39–5.95)	4.79 (4.33–5.22)	6.44 (4.96–9.17)	4.88 (4.30–5.5)	6.77 (4.96–9.54)	0.005
Creatinine (mg/dL)	6.28 (8.08–10.58)	8.38 (6.63–10.96)	7.09 (5.38–9.17)	8.32 (6.62–11.19)	6.87 (5.42–8.94)	0.535
Blood urea nitrogen (mmol/L)	17.41 (13.68–22.05)	17.8 (14.21–22.2)	17.43 (13.71–22.75)	17.43 (13.69–22.88)	15.21 (12.06–19.53)	0.021
Uric acid (umol/L)	398 (343–469)	388 (334–443.25)	383 (325.5–443.25)	420.50 (361.5–498)	403 (334.25–463.50)	<0.001
Total Cholesterol (mmol/L)	4.50 (3.88–5.17)	4.50 (4.19–5.31)	4.71 (4.26–5.43)	4.28 (3.53–4.84)	4.50 (3.78–5.28)	<0.001
Triglycerides (mmol/L)	1.38 (1.04–1.90)	1.35 (0.93–1.51)	1.34 (0.94–1.77)	1.46 (1.13–2.11)	1.65 (1.27–2.64)	<0.001
LDL (mmol/L)	2.57 (2.05–3.15)	2.63 (2.13–3.27)	2.71 (2.08–3.58)	2.42 (1.88–2.99)	2.57 (1.87–3.09)	<0.001
Calcium (mmol/L)	2.16 (2.02–2.27)	2.16 (2.05–2.28)	2.13 (1.94–2.21)	2.16 (2.02–2.25)	2.16 (2.02–2.25)	0.326
Phosphorus (mmol/L)	1.41 (1.15–1.77)	1.42 (1.16–1.74)	1.37 (1.18–1.70)	1.44 (1.16–1.86)	1.32 (1.10–1.53)	0.304
Potassium (mmol/L)	3.90 (3.43–4.41)	3.96 (3.48–4.43)	3.89 (3.44–4.44)	3.90 (3.47–4.44)	3.76 (3.31–4.31)	0.003
Outcome						
Pneumonia [*n* (%)]	137 (10.6%)	35 (6.5%)	23 (16.2%)	39 (8.8%)	40 (23.3%)	<0.001

BMI, body mass index; CVD, Cardiovascular disease; LDL, low density lipoprotein.

Group 0, patients without DM and low HDL-C; Group 1, patients with only DM; Group 2, patients with only low HDL-C; Group 3, patients with the coexistence of DM and low HDL-C.

The median age of the participants was 53 years, with a male preponderance of 57.3%. Additionally, 7.8% of the individuals reported a history of smoking. 4.3% of patients had pre-existing stroke, 22.75% of patients had pre-existing CVD, and 58.1% of patients had hypertension. There were no differences in drinking history, serum albumin, creatinine, serum calcium, serum phosphorus among four groups. The age, BMI, the proportion of smoking history, pre-existing stroke, pre-existing CVD and hypertension in the Group 3 were higher than those in other groups. The baseline laboratory results showed that Group 3 had higher levels of leukocytes, FBG, triglycerides, LDL-C, and lower levels of blood urea nitrogen and serum potassium (Table 1).

### Risk factors of new-onset pneumonia in PD patients

The risk factors of pneumonia in PD patients were explored by univariate Cox regression. Age, BMI, smoking history, pre-existing DM, pre-existing CVD, group 1, and group 3 were risk factors for pneumonia. Conversely, creatinine was protective factor (Table 2).

**Table 2:** Significant risk factors for new-onset pneumonia.

Variables	Univariate Cox analysis		
	HR	95%CI	*p*-value
Age (per 1-year greater)	1.028	1.015–1.041	<0.001
BMI (per 1-kg/m^2^ greater)	1.111	1.058–1.167	<0.001
Smoking history (yes vs. no)	1.821	1.162–2.854	0.009
DM (yes vs. no)	4.172	2.938–5.923	<0.001
Hypertension (yes vs. no)	7.346	4.455–12.112	<0.001
Pre-existing stroke (yes vs. no)	4.018	2.387–6.764	<0.001
Pre-existing CVD (yes vs. no)	3.612	2.480–5.261	<0.001
Creatinine (per1-mg/dL greater)	0.999	0.999–1.000	0.022
Group			
Group 0	1.000		
Group 1	3.214	1.882–5.489	<0.001
Group 2	0.909	0.573–1.440	0.684
Group 3	4.570	2.871–7.274	<0.001

BMI, body mass index; CVD, Cardiovascular disease; DM, diabetes mellitus.

Group 0, patients without DM and low HDL-C; Group 1, patients with only DM; Group 2, patients with only low HDL-C; Group 3, patients with the coexistence of DM and low HDL-C.

### DM coexisting low HDL-C and pneumonia

**Figure 3:**
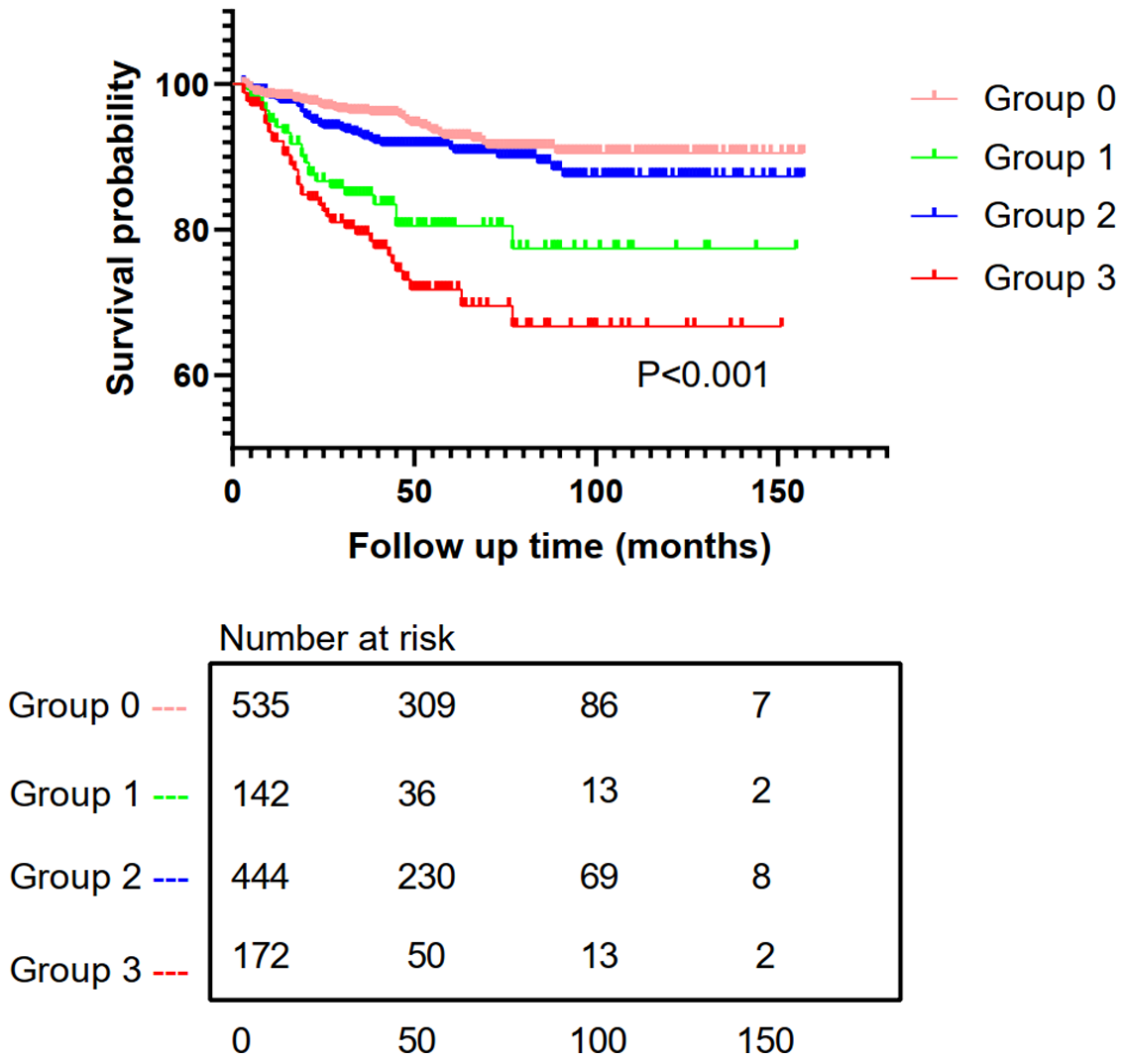
Kaplan–Meier survival analysis for the incidence of new-onset pneumonia. Group 0, patients without DM and low HDL-C; Group 1, patients with only DM; Group 2, patients with only low HDL-C; Group 3, patients with the coexistence of DM and low HDL-C.

Kaplan–Meier analysis demonstrated the cumulative incidence of new-onset pneumonia in Group 3 was significantly higher than that in the other three groups, with a statistically significant difference between groups (log-rank test: *p* < 0.001) (Figure 3).

Multivariable-adjusted Cox proportional hazards models revealed significantly increased pneumonia risk in Group 3 compared with Group 0 (HR = 2.252, 95% CI, 1.232–4.115) (Table 3). PD patients with coexisting DM and low HDL-C had a 2.252-fold higher risk of developing pneumonia than those without either condition.

**Table 3:** Association among DM and low HDL-C and the new-onset pneumonia.

Variables	Model 1		Model 2		Model 3	
	HR	95% CI	HR	95% CI	HR	95% CI
Group 0	1.0 (ref.)		1.0 (ref.)		1.0 (ref.)	
Group 1	3.214*	1.882–5.489	1.437	0.823–2.509	1.672	0.905–3.090
Group 2	0.909	0.573–1.440	0.927	0.578–1.487	1.133	0.653–1.964
Group 3	4.570*	2.871–7.274	1.794*	1.075-2.995	2.252*	1.232-4.115

Model 1: Unadjusted.

Model 2: adjusted age, sex, BMI, smoking, pre-existing stroke, pre-existing CVD, pre-existing hypertension.

Model 3: Model 2 plus hemoglobin, serum albumin, FBG, cholesterol, triglyceride, LDL-C, creatinine, uric acid, serum potassium, serum calcium.

^*^p < 0.05.

Group 0, patients without DM and low HDL-C; Group 1, patients with only DM; Group 2, patients with only low HDL-C; Group 3, patients with the coexistence of DM and low HDL-C.

### Subgroup analyses

**Figure 4:**
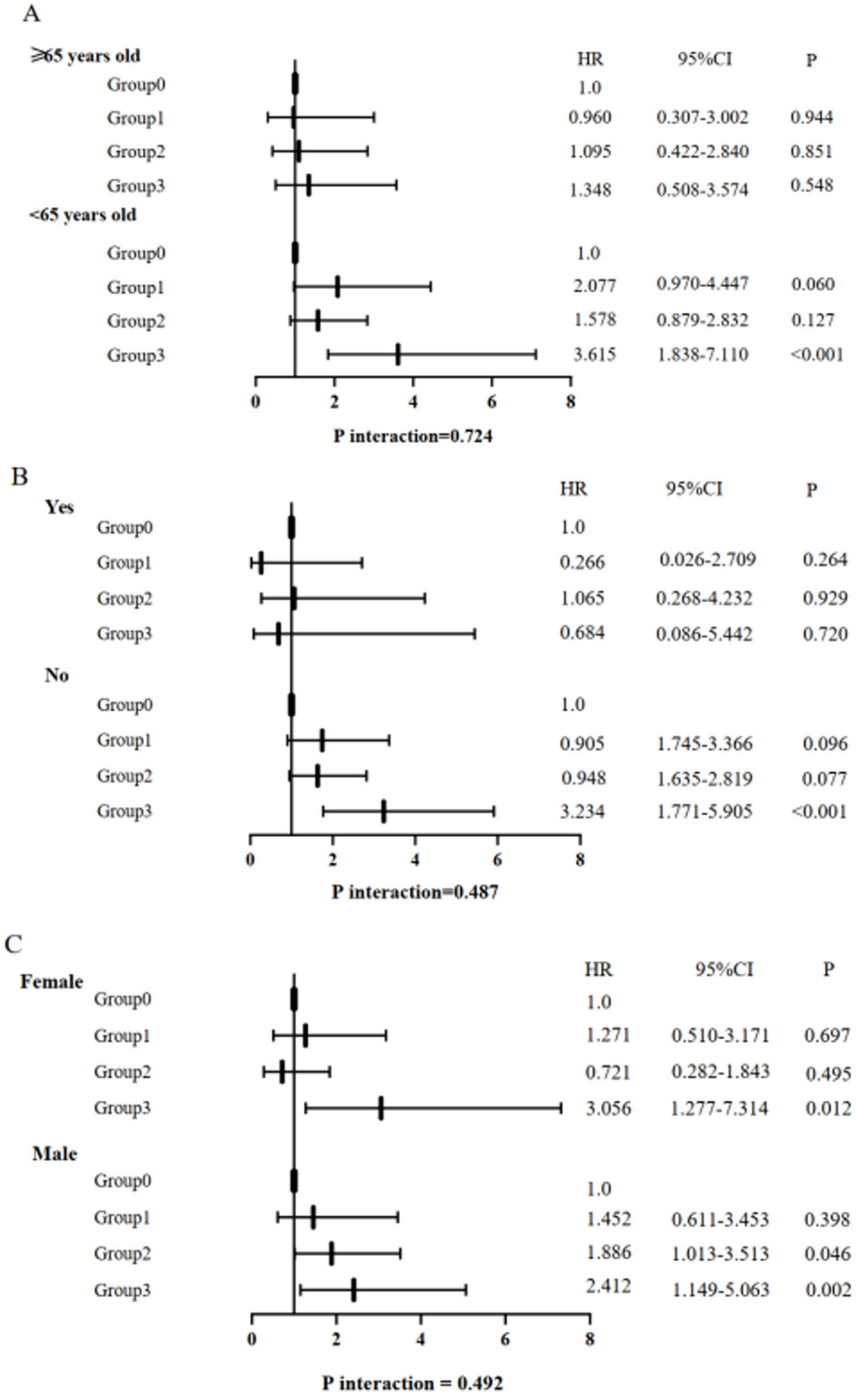
Association between diabetes mellitus and low HDL cholesterol and pneumonia between subgroups. **(A)** Age group; **(B)** smoking history group; **(C)** sex group. Group 0, patients without DM and low HDL-C. Group 1, patients with only DM. Group 2, patients with only low HDL-C. Group 3, patients with the coexistence of DM and low HDL-C.

Subgroup interaction analyses demonstrated non-significant modification effects across age (*p* = 0.724), sex (*p* = 0.492), and smoking history (*p* = 0.487) stratifications. This suggested that regardless of these variables, the increased risk of pneumonia associated with the coexistence of DM and low HDL-C is significant. And the risk of pneumonia in Group 3 was relatively high in all subgroups (Figure 4).

## Discussion

This multicenter retrospective study found that 10.6% of PD patients developed pneumonia during PD treatment and the coexistence of DM and low HDL-C at baseline was associated with an increased risk of new-onset pneumonia.

Glucose-based PD solution affects lipid and glucose metabolism in PD patients. DM and low HDL-C levels interact in PD patients. This highlights the importance of increased vigilance regarding the risk of infection in this population. In patients with DM, downregulation of the apolipoprotein A-I gene and LCAT mRNA results in reduced plasma apolipoprotein A-I levels and LCAT activity, thereby decreasing HDL-C (15). Low levels of HDL-C can affect the function and survival of islet *β* cells (16). HDL-C can reduce stress-induced islet β-cell apoptosis, increase insulin secretion, and mitigate inflammation in DM via ATP-binding cassette transporters A1 and G1 (ABCA1 and ABCG1) (17). DM is associated with various infectious diseases (18, 19). In a study of the general population, after adjusting for potential confounding factors, DM was found to be associated with a 1.75-fold higher risk of pneumonia compared with non-diabetic patients (5). This is consistent with our study, where PD patients with DM alone have a higher risk of developing pneumonia than those without DM. This may be related to the low systemic immune function of patients with DM, including the low activity of NK cells and the increase of inflammatory cytokines (20, 21). Hyperglycemia induces inflammation and structural changes in lung parenchyma and vasculature (22). Exposure to elevated glucose concentration in lung epithelial cells increases the risk of pathogen infection and replication (23).

Decreased HDL-C levels can impair its anti-inflammatory function and increase the risk of infection (24, 25). Numerous studies have consistently linked low HDL-C to infectious diseases. In a longitudinal cohort study, lower baseline HDL-C was strongly correlated with an elevated risk of long-term pneumonia hospitalization (11). Low HDL-C levels are associated with an increased risk of foot ulceration in DM patients (26). Additionally, Grion et al. demonstrated that reduced HDL-C may be a risk factor for severe sepsis in hospitalized patients (27). HDL-C is recognized for its crucial role in the innate immune response, effectively combating pathogens by rapidly inducing an oxidative state. It also demonstrates anti-inflammatory properties by impacting the expression of local adhesion molecules and the secretion of cytokines by immune cells (28). Therefore, it is crucial to maintain normal serum HDL-C levels in patients with DM undergoing PD.

Several risk factors for pneumonia in dialysis patients have been reported, including senior, immobility, chronic obstructive pulmonary disease (COPD), smoking history, DM, obesity, and hypoalbuminemia (3, 6). This is consistent with the results obtained in our study, and similar conclusions are obtained in univariate Cox regression. Patients with DM combined with low HDL-C were older, had higher BMI, and a higher proportion of smokers than the other groups. These findings suggested a possible link between metabolic syndrome and inflammatory status. Obesity increases patients’ risk of developing DM and is associated with higher mortality (29).

This study is subject to several important limitations. First, as a retrospective study, although potential baseline confounding variables were adjusted, it was not possible to determine the causal relationship between the coexistence of DM and low HDL-C and pneumonia. Second, this study only included patients with type 2 DM, thus it remains unclear whether the coexistence of type 1 DM and low HDL-C is associated with an increased risk of new-onset pneumonia. And the absence of glycosylated hemoglobin testing during follow-up precluded evaluation of long-term glycemic control in these DM patients. Third, only baseline blood lipid levels were recorded, so a large-scale prospective follow-up study is needed to explore blood lipid changes’ impact on pneumonia occurrence. Fourth, since all participants were recruited from five Chinese PD centers, the generalizability of our findings to wider clinical practice may be limited. Finally, due to retrospective study limitations, data on catheter-related factors, vaccination status, baseline respiratory comorbidities, and glucose-lowering/lipid-lowering medications were not captured, which may serve as the primary source of residual confounding in this study.

## Conclusion

This study suggests that PD patients with coexisting DM and low HDL-C face a significantly higher risk of new-onset pneumonia compared to those with neither condition or to those with DM alone and normal HDL-C. In PD patients with DM, higher HDL-C levels may be associated with a lower risk of infection. Monitoring and management of HDL-C should be strengthened for PD patients with DM.

## Data Availability

The datasets generated and analyzed during the current study are not publicly available due to patient confidentiality and privacy agreements. Data sharing will be subject to the approval of the institutional ethics committee and the execution of a data use agreement. Requests to access these datasets should be directed to NS, suning5@mail.sysu.edu.cn.
